# Dissociations between Learning Phenomena do Not Necessitate Multiple Learning Processes: Mere Instructions about Upcoming Stimulus Presentations Differentially Influence Liking and Expectancy

**DOI:** 10.5334/joc.59

**Published:** 2019-03-01

**Authors:** Jan De Houwer, Simone Mattavelli, Pieter Van Dessel

**Affiliations:** 1 Ghent University, BE

**Keywords:** Learning, Emotion and cognition, Memory

## Abstract

Prior research showed that the degree of statistical contingency between the presence of stimuli moderates changes in expectancies about the presence of those stimuli (i.e., expectancy learning) but not changes in the liking of those stimuli (i.e., evaluative conditioning). This dissociation is typically interpreted as evidence for dual process models of associative learning. We tested an alternative account according to which both types of learning rely on a single process propositional learning mechanism but reflect different kinds of propositional beliefs. In line with the idea that changes in liking reflect beliefs about stimulus co-occurrences whereas changes in expectancy reflect beliefs about stimulus contingency, we found that evaluative ratings depended only on instructions about whether a stimulus would co-occur with a positive or negative stimulus whereas expectancy ratings were influenced also by instructions about individual stimulus presentations.

When trying to map the mental processes that mediate behavior, cognitive psychologists often rely on dissociations, that is, the differential impact of experimental manipulations on different behavioral phenomena (see Dunn & Kirsner, 2003, for a succinct discussion). In the current paper, we focus on one such dissociation in research on learning, namely the observation that statistical contingency matters for expectancy learning but not for evaluative conditioning. Evaluative conditioning refers to the impact of stimulus pairings on liking (De Houwer, 2007; see Hofmann, De Houwer, Perugini, Baeyens, & Crombez, 2010, for a review and meta-analysis). For instance, pairing a novel brand name with attractive images tends to increase the liking of that brand name (e.g., Pleyers, Corneille, Luminet, & Yzerbyt, 2007). Expectancy learning can be defined as the impact of stimulus pairings on the extent to which the presence of one of these stimuli generates an expectancy of the presence of the other stimulus (e.g., Hermans, Vansteenwegen, Crombez, Baeyens, & Eelen, 2002). For instance, after pairing the picture of a human face and an electric shock, presentation of the face leads to an expectancy of the shock as indicated by ratings or physiological responses (e.g., skin conductance; Hermans et al., 2002; Vansteenwegen, Francken, Vervliet, De Clercq, & Eelen, 2006). Both types of learning thus involve effects of stimulus pairings (i.e., they are both instances of associative learning) but differ with regard to the variable that changes as the result of the pairings (i.e., liking vs. expectancy).

Despite the fact that evaluative conditioning and expectancy learning are quite similar at the procedural level, it has been argued that they are fundamentally different at the mental process level. According to referential learning theory (Baeyens, Eelen, Van den Bergh, & Crombez, 1992; Sweldens, Van Osselaer, & Janiszewski, 2010), evaluative conditioning is an instance of referential learning that relies on a contiguity-driven formation of associations in memory (i.e., a Hebbian-like “what fires together wires together” mechanism). Expectancy learning, on the other hand, is assumed to rely on more complex association formation processes that take into account not only stimulus co-occurrences but also events during which a stimulus occurs in isolation. This dual process view on learning predicts that one should be able to dissociate evaluative conditioning and expectancy learning, most prominently with regard to the impact of statistical contingency, that is, the relative frequency with which stimuli are present together, present on their own, or absent together. More specifically, expectancy learning should reflect the degree of statistical contingency whereas evaluative conditioning should reflect only whether and how often stimuli co-occur.

Unlike other claims about dissociations between evaluative conditioning and expectancy learning (e.g., that evaluative conditioning but not expectancy learning can occur unconsciously; e.g., Baeyens, Eelen, & Van den Bergh, 1990; see Corneille & Stahl, in press, for a discussion), the claim that statistical contingency has a different impact on expectancy learning than on evaluative conditioning is relatively undisputed. Ever since the studies of Rescorla (1966), there is little doubt that statistical contingency is vital for most types of associative learning, including expectancy learning. In evaluative conditioning research, on the other hand, little evidence has been found for an impact of statistical contingency (Baeyens, Hermans, & Eelen, 1993; Hofmann et al., 2010; Kattner, 2014; but see Halbeisen & Walther, 2016). Instead, there is some (albeit weak) evidence that evaluative conditioning depends primarily on the number of stimulus co-occurrences (Hofmann et al., 2010; Kattner, 2014). In sum, as predicted by referential learning theory, prior research shows that statistical contingency differentially influences evaluative conditioning and expectancy learning.

Logic dictates, however, that the affirmation of a theoretical prediction does not necessitate the validity of the theory because there could be other theories that make the same prediction (i.e., the fallacy of affirming the consequent). This consideration is relevant also in the context of the present paper. Hofmann et al. (2010) pointed out that statistical contingency might have a different effect on evaluative conditioning than on expectancy learning even when both types of learning depend on a single propositional learning system. According to propositional theories of associative learning (De Houwer, 2009, 2018; Mitchell, De Houwer, & Lovibond, 2009), stimulus pairings can have an impact on behavior only after a propositional belief has been formed about the relation between the stimuli. As De Houwer (2009, pp. 6–7) pointed out, different propositions can be formed about the same stimulus relation. For instance, assume that a conditioned stimulus (CS; e.g., a novel brand name or tone) and unconditioned stimulus (US; e.g., a positive picture or an electric shock) co-occur on 8 trials and that the CS is presented on its own on 8 other trials. Based on these events, participants could form both the belief that “the CS and US sometimes co-occur” (i.e., a co-occurrence belief) as well as the belief that “the CS is a poor predictor of the US” (i.e., a contingency belief). Both beliefs are valid and do not contradict each other. Whereas the former belief should be influenced only by whether the CS and US actually co-occur (e.g., the 8 CS-US trials), the latter belief would take into account also the number of trials on which the CS is presented without the US. Hence, if the impact of stimulus pairings on liking (i.e., evaluative conditioning) is mediated by a co-occurrence belief and the impact of stimulus pairings on US expectancy (i.e., expectancy learning) is mediated by a contingency belief, then a manipulation of stimulus contingency (e.g., keeping constant the number of CS-US co-occurrences while varying the number of CS-only trials) should have an impact on expectancy learning but not evaluative conditioning.

Having an alternative, post-hoc explanation for a dissociation of course does not mean that the original explanation is wrong and that the alternative one is correct. On the contrary, post-hoc theories should be taken seriously only to the extent that they generate novel predictions that can be verified empirically. Fortunately, novel predictions can be derived from the propositional account of the dissociation that we have focused on in this paper. Propositional theories of learning advocate that beliefs about stimulus relations can be formed not only on the basis of the actual presence or absence of stimuli but also on the basis of instructions about the presence and absence of those stimuli (De Houwer, 2009). Hence, if the dissociation results from the fact that evaluative conditioning and expectancy learning are mediated by different types of propositional beliefs, then a dissociation should arise also as the result of an instruction-based manipulation of those beliefs, even when there a no actual stimulus pairings. Although there is abundant evidence that mere instructions about stimulus pairings are sufficient to produce changes in liking and expectancies (e.g., Cook & Harris, 1937; Gast & De Houwer, 2013; Mertens, Boddez, Sevenster, Engelhard, & De Houwer, 2018), it has not yet been tested whether a manipulation of the content of these instructions can have a differential impact on changes in liking and expectancies.

We examined this issue by telling participants that, on each trial of an upcoming learning phase, they would see one of two nonwords. A first nonword (CS+) would sometimes be followed by an aversive scream (US); another nonword (CS–) would never be followed by the scream. Depending on the condition to which they were assigned, participants learned that the CS+ nonword would be (a) paired with the US scream on 8 trials and presented on its own on 2 trials (high contingency, high co-occurrence condition), (b) paired with the US on 8 trials and presented alone on 8 trials (low contingency, high co-occurrence condition), (c) paired with the US on 4 trials and presented alone on 1 trial (high contingency, low co-occurrence), or (d) paired with the US on 4 trials and presented alone on 4 trials (low contingency, low co-occurrence). Hence, in addition to manipulating contingency information (whether the instructed events implied a high or low statistical contingency), we also manipulated information about the number of co-occurrences, thus allowing us to examine the impact of both types of information.

After verifying that participants had understood the instructions, they experienced only two trials, one in which the CS+ was presented and one in which the CS– was presented, but neither of which contained the US. On each trial, they first saw the CS and then rated the extent to which they expected the US to occur on that trial and the extent to which they liked that CS (order of ratings counterbalanced between participants). After both ratings were entered, participants were informed that a US would not be presented in that trial. After the second trial, they were told that no additional learning trials would be presented and were asked to complete a reaction time task. This task was an Implicit Association Test (IAT; Greenwald, McGhee, & Schwartz, 1998) that was designed to capture implicit (i.e., spontaneous) evaluations of the CSs. IATs are often used in evaluative conditioning research as a way of complementing evaluative ratings that typically capture more well-reasoned and thus less spontaneous evaluations of stimuli (see Hofmann et al., 2010, for a review). We therefore also included an IAT measure in our study even though we did not have specific predictions about differences in the results on these two measures of stimulus evaluation.^1^ Based on propositional theories of associative learning, our main prediction was that the manipulation of information about stimulus contingency would influence changes in US expectancy but not changes in CS liking. To test this prediction, we conducted two experiments, the second of which aimed to verify the results of the first.

## Method

### Participants

A total of 200 and 220 English-speaking volunteers participated online via the Prolific Academic website (https://prolific.ac) in Experiments 1 and 2, respectively. Sample size was determined based on an a priori power analysis such that we would have sufficient power (i.e., power > 0.94) to detect a moderate between-subjects effect (*d* = 0.50). Prior to data-collection, target sample size was pre-registered together with the study design, data-analytic plans, and experimental hypotheses. The pre-registered plans, raw data, experimental and analytic scripts are available at https://osf.io/f9jn6/.

The experiment was programmed in Inquisit 4.0 and hosted via Inquisit Web (Millisecond Software, Seattle, WA). Following our pre-registered data analysis plan, we excluded the data from participants who (a) did not fully complete all questions and tasks (Experiment 1: 13 participants; i.e., 6.50%; Experiment 2: 6 participants; i.e., 2.73%) or (b) reported that they did not hear an auditory stimulus after its presentation (Experiment 1: 6 participants; i.e., 2.00%; Experiment 2: 6 participants; i.e., 2.73%). Analyses were performed on the data of 181 participants in Experiment 1 (113 women, mean age = 34, *SD* = 12) and 208 participants in Experiment 2 (119 women, mean age = 37, *SD* = 11).

### Materials

The US was selected on the basis of a rating study in which an on-line sample of 34 Prolific Academic participants rated the averseness of 15 four-second auditory stimuli (each presented twice) on a 21-points scale ranging from –10 (very negative) to +10 (very positive). We selected the sound of a woman’s scream because this auditory stimulus was judged as the most aversive (*M* = 7.65, *SD* = 2.96). Two nonsense words (MORAG and STRUAN) were used as CSs. It was counterbalanced across participants which word served as CS+ or CS–.

### Procedure

**Experiment 1.** Participants first provided informed consent and answered demographic questions asking for their age, gender, and country of residence. They were then told that the experiment would involve both visual and auditory stimuli and they were instructed to put on their headphones and set the sound volume to 30%. Next, participants were asked to listen to the auditory stimulus and to briefly describe it.

Participants then received instructions which specified that two words (MORAG and STRUAN) would be presented on screen several times and that they would sometimes be followed by the sound they had heard previously. There were four different between-subjects instruction conditions, formed by crossing instructed US-CS+ contingency (high: 80% vs. low: 50%) and instructed US-CS+ co-occurrence (high: 8 trials vs. low: 4 trials). Specifically, participants were informed that one word (the CS+) would be presented either for (a) 10 trials, 8 times followed by the sound and 2 times not (high contingency, high co-occurrence), (b) 16 trials, 8 times followed by the sound and 8 times not (low contingency, high co-occurrence), (c) 5 trials, 4 times followed by the sound and once not (high contingency, low co-occurrence), and (d) 8 trials, 4 times followed by the sound and 4 times not (low contingency, low co-occurrence). All participants were told that the other word (the CS–) would never be followed by the sound. To check whether the participants understood the instructions, they were then asked to indicate how often each of the two words would and would not be followed by the sound. Participants could proceed to the main task only if they were able to remember the correct number of events for both words (e.g., MORAG: 4 times followed by the sound, 4 times not followed; STRUAN: never followed by the sound). Otherwise, participants saw the instruction screen again.

After the retention test, participants were informed that they would now perform the task in which the two CSs would be presented in the center of the screen for 4 seconds either followed by the sound or not. Participants completed two trials, one in which the CS+ was presented and one in which the CS– was presented. After each presentation, participants saw two rating scales (in counterbalanced order), asking them to rate (a) the extent to which they expected the sound on a 9-point Likert scale ranging from 1 (not at all) to 9 (certainly) or (b) their liking of the CS on a 21-point Likert scale ranging from –10 (very negative) to +10 (very positive). After providing the two ratings, participants saw a prompt that there was no sound on this occasion. After completing both trials, participants were informed that they had been assigned to a condition in which only a single trial was presented for each word.

Participants then completed an IAT that measured implicit evaluations of the two CSs. Participants categorized eight attribute words (e.g., wonderful, awful) as ‘positive’ or ‘negative’ and the two CSs as their respective names (‘Struan’ and ‘Morag’). To avoid that the CSs were classified only on the basis of simple perceptual features, we presented them in different font types (Arial Black and Fixedsys) and capitalizations (uppercase and lowercase). The attribute words were always presented in Arial Black, font size 16, lower case. The IATs consisted of three practice blocks and two experimental blocks. Participants began with 16 practice trials sorting the CSs and 16 practice trials sorting the attribute words. Next, participants completed 64 trials in which the CS+ and positive words shared one response key and the CS– and negative words shared another response key (or the opposite combination of CS type and valence). IAT response mapping was orthogonally crossed with instruction condition. Participants then practiced sorting the CSs on 16 trials with the reversed response key assignment. Finally, participants completed a second set of 64 trials in which the CS+ now shared a response key with negative and the CS– shared a response key with positive (or vice versa). On each trial, a word or CS was presented in the center of the screen until the participant pressed one of the two valid keys (i.e., ‘E’ or ‘I’). If the response was correct, the stimulus disappeared and the next stimulus was presented 400ms later. If the response was incorrect, the stimulus was replaced by a red ‘X’ for 200ms and participants had to correct their mistake to continue to the next trial.

After completion of the IAT, participants first provided liking and US expectancy ratings of the two USs for a second time (Time 2 measurement). Specifically, participants were asked to think back to the part of the study immediately after they received instructions about the presentation frequency of the two words. They rated to what extent they expected the sound after each CS presentation and how much they liked each CS. Rating scales and measurement order were identical to Time 1 measurement. Participants then rated the pleasantness of the US on an 11-point Likert scale ranging from –5 (unpleasant) to +5 (pleasant). Next, participants indicated the clarity and believability of the instructions on a 10-point Likert scale and were asked to provide general remarks about the experiment. Finally, participants answered the same questions about CS-US contingencies as in the retention test. Exclusion from analyses of participants who gave an incorrect response (Experiment 1: 14 participants; i.e., 7.65%; Experiment 2: 20 participants; i.e., 9.62%) did not change the significance level of any of the reported findings.

**Experiment 2.** The main aim of Experiment 2 was to verify the results of Experiment 1. The procedure of Experiment 2 was therefore identical to that of Experiment 1. The only exception was that, for exploratory reasons, we added a third measurement immediately after the Time 2 measurement. In this Time 3 measure, participants were asked to indicate how they felt about the stimulus at the current time and to what extent they would expect the sound if the CS would now be presented. Hence, whereas for the Time 2 measurements, participants were asked to report the feelings and beliefs they had immediately after receiving the instructions about the stimulus presentations, for the Time 3 measurement, participants were asked to report their feelings and beliefs at that time, that is, at the end of the experiment.

## Results

### Experiment 1

**US expectancy ratings.** We performed a mixed ANOVA with CS Type (CS+, CS–) and Time (Time 1, Time 2) as within-subjects factors and Contingency (high: 80%, low: 20%) and Co-occurrence (high: 8 trials, low: 4 trials) as between-subjects factors. There was a main effect of Contingency, *F*(1, 177) = 17.82, *p* < .001, *η^2^* = .09, indicating higher US expectancy ratings when the instructed contingency was high (*M* = 4.13, *SD* = 3.17) than when it was low (*M* = 3.47, *SD* = 2.46) (Table 1). A Bayes factor was calculated according to the procedures outlined by Rouder, Speckman, Sun, Morey, and Iverson (2009) that gives an indication of how strongly the data support either the null hypothesis (BF_0_; reflecting the absence of a significant effect) or the alternative hypothesis (BF_1_; reflecting the presence of a significant effect). The Bayes factor indicates strong evidence for the presence of the main effect of Contingency, BF_1_ = 490.53. We also observed a main effect of CS Type, *F*(1, 177) = 387.78, *p* < .001, *η^2^* = .69, indicating higher US expectancy ratings for the CS+ (*M* = 5.82, *SD* = 2.28) than for the CS– (*M* = 1.78, *SD* = 1.78), BF_1_ > 10^5^. Most importantly, the interaction between CS Type and Contingency was also significant, *F*(1, 177) = 7.37, *p* = .007, *η^2^* = .04. As predicted, the effect of CS Type (higher expectancy ratings for CS+ than for CS–) was larger when the instructed contingency was high (CS+: *M* = 6.43, *SD* = 2.47; CS–: *M* = 1.83, *SD* = 1.85), *t*(179) = 17.36, *p* < .001, *d* = 1.30, than when it was low (CS+: *M* = 5.22, *SD* = 1.80; CS–: *M* = 1.73, *SD* = 1.69), *t*(179) = 11.11, *p* < .001, *d* = 0.83. The Bayes factor indicates substantial evidence for the presence of this effect, BF_1_ = 5.03. Finally, we also observed an interaction effect of CS Type and Time, *F*(1, 177) = 6.40, *p* = .010, *η^2^* = .03, indicating a smaller effect of CS Type at Time 1 (CS+: *M* = 5.66, *SD* = 2.32; CS–: *M* = 1.88, *SD* = 1.91), *t*(179) = 16.56, *p* < .001, *d* = 1.23, BF_1_ > 10^5^, than at Time 2 (CS+: *M* = 5.98, *SD* = 2.23; CS–: *M* = 1.69, *SD* = 1.65), *t*(179) = 18.79, *p* < .001, *d* = 1.40, BF_1_ > 10^5^. However, the evidence for this interaction effect was only anecdotal, BF_1_ = 1.83. None of the effects that included the Co-occurrence factor were significant, *F*s < 1.34, *p*s > .25, *η^2^*s < .01, BF_1_s < 1.24.

**Table 1:** Means and standard deviations of US expectancy and liking ratings in Experiment 1.

		US expectancy ratings		Liking ratings	

		CS+	CS–	CS+	CS–

**Time 1**					
Contingency high	Co-occurrence high	6.38 (2.40)	1.94 (2.04)	–1.32 (5.03)	0.66 (5.21)
	Co-occurrence low	6.07 (2.68)	1.83 (1.98)	–1.02 (4.42)	0.78 (4.27)
Contingency low	Co-occurrence high	4.96 (2.05)	1.72 (1.61)	–0.98 (4.97)	1.57 (5.78)
	Co-occurrence low	5.06 (1.44)	1.84 (1.79)	–2.03 (3.33)	0.29 (3.93)
**Time 2**					
Contingency high	Co-occurrence high	6.42 (2.48)	1.78 (1.81)	–1.04 (5.26)	2.18 (4.03)
	Co-occurrence low	6.67 (2.33)	1.59 (1.60)	–0.61 (4.88)	0.54 (4.21)
Contingency low	Co-occurrence high	5.35 (2.02)	1.46 (1.28)	–0.59 (4.54)	2.11 (4.64)
	Co-occurrence low	5.32 (1.47)	1.74 (1.75)	0.16 (3.79)	0.35 (4.39)

**Liking ratings.** The same mixed ANOVA on liking ratings revealed a main effect of CS Type, *F*(1, 177) = 29.31, *p* < .001, *η^2^* = .14, indicating lower liking ratings for the CS+ (*M* = –0.90, *SD* = 4.64) than for the CS– (*M* = 1.08, *SD* = 4.62), BF_1_ > 10^5^, and a main effect of Time, *F*(1, 177) = 6.78, *p* = .010, *η^2^* = .04, indicating higher liking ratings at Time 2 (*M* = 0.41, *SD* = 4.62) than at Time 1 (*M* = –0.23, *SD* = 4.84), BF_1_ = 2.20. In contrast to the analysis of the US expectancy ratings, we did not observe a significant interaction between CS Type and Contingency, *F*(1, 177) = 0.02, *p* = .88, *η^2^* < .01, with the Bayes Factor indicating substantial evidence for the absence of this effect, BF_0_ = 6.11, nor any other effects involving Contingency, *F*s < 2.24, *p*s > .14, *η^2^*s < .01, BF_0_s > 3.66. We did observe a three-way interaction between Co-occurrence, CS Type, and Time, *F*(1, 179) = 4.72, *p* = .030, *η^2^* = .03. This interaction indicated that, for Time 2 ratings, a larger effect of CS Type was observed when instructed Co-occurrence was high, *t*(95) = 4.53, *p* < .001, BF_1_ = 955.96, than when it was low, *t*(84) = 1.08, *p* = .28, BF_0_ = 4.75, but this differential effect was not observed for Time 1 ratings (high instructed Co-occurrence: *t*[*95*] = 4.60, *p* < .001, BF_1_ = 1225.75; low instructed Co-occurrence: *t*[*84*] = 3.95, *p* < .001, BF_1_ = 124.76).

**IAT.** In-line with our data analysis plan, we excluded the IAT data of participants who had error rates above 30% across the entire task or above 40% for any one of the four critical IAT blocks or completed more than 10% of IAT trials faster than 400ms (Experiment 1: 10 participants; i.e., 5.46%; Experiment 2: 7 participants; i.e., 5.39%). IAT scores were calculated using the D2-algorithm (Greenwald, Nosek, & Banaji, 2003) so that a positive score indicates a preference for the CS+ over the CS–. A 2 (Contingency) × 2 (Co-occurrence) ANOVA revealed a significant effect of Contingency, *F*(1, 167) = 4.21, *p* = .042, *η^2^* = .03, BF_1_ = 1.58. IAT scores were significantly higher than zero when instructed contingency was low (*M* = 0.14, *SD* = 0.39), *t*(71) = 3.12, *p* = .003, *d* = 0.37, BF_1_ = 10.50, but not when it was high (*M* = 0.00, *SD* = 0.42), *t*(98) = 0.10, *p* = .92, *d* = 0.01, BF_0_ = 8.95. This indicates that, contrary to expectations, participants liked the CS+ more than the CS– but only when contingency was low. We did not observe any other effects, *F*s < 0.81, *p*s > .37, *η^2^*s < .01, BF_0_s > 3.27.

### Experiment 2

**US expectancy ratings.** We again observed a main effect of Contingency, *F*(1, 204) = 26.22, *p* < .001, *η^2^* = .11, indicating higher US expectancy ratings when the instructed contingency was high *M* = 4.47, *SD* = 3.18) than when it was low (*M* = 3.64, *SD* = 2.49), BF_1_ > 10^5^ (Table 2) and a main effect of CS Type, *F*(1, 204) = 567.71, *p* < .001, *η^2^* = .74, indicating higher US expectancy ratings for the CS+ (*M* = 6.03, *SD* = 2.05) than for the CS– (*M* = 2.07, *SD* = 2.17), BF_1_ > 10^5^. There was also a main effect of Time, *F*(2, 408) = 4.08, *p* = .018, *η^2^* = .02, indicating higher US expectancy ratings at Time 3 (*M* = 4.22, *SD* = 2.85) than at Time 1 (*M* = 3.95, *SD* = 2.94), *t*(207) = 2.63, *p* = .024, BF_1_ = 2.22, but not compared to Time 2 (*M* = 3.99, *SD* = 2.91), *t*(207) = 2.27, *p* = .061, BF_0_ =1.05. Most importantly, we again observed an interaction effect of Contingency and CS Type, *F*(1, 204) = 7.82, *p* = .006, *η^2^* = .04. The effect of CS Type was larger when the instructed contingency was high (CS+: *M* = 6.68, *SD* = 2.11; CS–: *M* = 2.26, *SD* = 2.45), *t*(207) = 19.20, *p* < .001, *d* = 1.32, BF_1_ > 10^5^, than when it was low (CS+: *M* = 5.39, *SD* = 1.74; CS–: *M* = 1.89, *SD* = 1.79), *t*(157) = 14.59, *p* < .001, *d* = 1.02, BF_1_ > 10^5^. The Bayes factor indicates substantial evidence for the presence of this interaction effect, BF_1_ = 5.67. None of the effects that included the Co-occurrence factor were significant, *F*s < 2.02, *p*s > .15, *η^2^*s < .01, BF_0_s > 2.63.

**Table 2:** Means and standard deviations of US expectancy and liking ratings in Experiment 2.

		US expectancy ratings		Liking ratings	

		CS+	CS–	CS+	CS–

**Time 1**					
Contingency high	Co-occurrence high	6.48 (2.30)	2.07 (2.52)	–0.87 (5.35)	–0.04 (5.92)
	Co-occurrence low	6.57 (2.19)	2.04 (2.15)	0.28 (4.60)	1.80 (4.18)
Contingency low	Co-occurrence high	5.31 (1.69)	1.51 (1.46)	–1.00 (4.56)	0.65 (4.44)
	Co-occurrence low	5.63 (1.80)	1.90 (2.13)	–0.39 (4.59)	1.20 (5.27)
**Time 2**					
Contingency high	Co-occurrence high	6.78 (2.13)	2.00 (2.40)	–0.30 (5.17)	2.17 (4.76)
	Co-occurrence low	6.57 (2.14)	2.41 (2.54)	0.50 (4.29)	2.28 (3.44)
Contingency low	Co-occurrence high	5.27 (1.67)	1.43 (1.08)	–0.75 (3.81)	1.76 (4.41)
	Co-occurrence low	5.39 (1.91)	1.96 (1.79)	–0.06 (4.16)	1.73 (4.88)
**Time 3**					
Contingency high	Co-occurrence high	6.78 (2.15)	2.26 (2.36)	0.00 (5.34)	1.98 (4.64)
	Co-occurrence low	6.83 (1.80)	2.69 (2.75)	0.78 (4.12)	1.80 (3.83)
Contingency low	Co-occurrence high	5.31 (1.59)	1.88 (1.62)	–0.35 (4.01)	1.37 (4.25)
	Co-occurrence low	5.31 (1.84)	2.57 (2.31)	0.10 (4.22)	1.80 (1.48)

**Liking ratings.** The ANOVA revealed effects of CS Type, *F*(1, 204) = 32.81, *p* < .001, *η^2^* = .14, indicating lower liking ratings for the CS+ (*M* = –0.17, *SD* = 4.60) than for the CS– (*M* = 1.56, *SD* = 4.58), BF_1_ > 10^5^, and Time, *F*(2,408) = 8.15, *p* < .001, *η^2^* = .05, indicating lower liking ratings at Time 1 (*M* = 0.21, *SD* = 2.85) than at Time 2 (*M* = 0.92, *SD* = 2.94) or Time 3 (*M* = 0.97, *SD* = 2.91), *t*s > 3.38, *p*s < .003, BF_1_s > 38.03. More importantly, however, we did not observe an interaction between CS Type and Contingency, *F*(1, 204) = 0.19, *p* = .66, *η^2^* < .01, BF_0_ = 6.04, nor any other effects of Contingency, *F*s < 1.00, *p*s > .34, *η^2^*s < .01, BF_0_s > 17.58, or Co-occurrence, *F*s < 1.85, *p*s > .17, *η^2^*s < .01, BF_0_s > 0.99.

**IAT.** The ANOVA revealed only a marginally significant effect of Contingency, *F*(1, 197) = 3.72, *p* = .055, *η^2^* = .02, BF_0_ = 1.17. IAT scores were significantly higher than zero when instructed contingency was low (*M* = 0.12, *SD* = 0.44), *t*(95) = 2.66, *p* = .009, *d* = 0.27, BF_1_ = 3.15, but not when it was high (*M* = –0.01, *SD* = 0.51), *t*(104) = –0.20, *p* = .84, *d* = 0.02, BF_0_ = 9.07. We did not observe any other effects, *F*s < 0.95, *p*s > .33, *η^2^*s < .01, BF_0_s > 4.28.

### Experiments combined

**US expectancy and liking ratings.** To directly compare changes in expectancy and liking in a test with sufficient power, we performed an ANOVA on standardized Time 1 and Time 2 US expectancy and liking ratings of Experiments 1 and 2 with CS Type, Time, Contingency, Co-occurrence and Measure (US Expectancy, Liking) as factors. The ANOVA revealed a main effect of CS Type, *F*(1, 385) = 274.89, *p* < .001, *η^2^* = .42, BF_1_ > 10^5^, which was qualified by an interaction of CS Type and Measure, *F*(1, 385) = 575.93, *p* < .001, *η^2^* = .60, BF_1_ > 10^5^. US expectancy ratings were higher for the CS+ than for the CS–, *p* < .001, *d* = 2.00, BF_1_ > 10^5^ whereas liking ratings were lower for the CS+ than for the CS–, *p* < .001, *d* = 0.41, BF_1_ > 10^5^. We also observed a main effect of Contingency, *F*(1, 385) = 9.87, *p* = .002, *η^2^* = .02, BF_1_ > 10^5^, and an interaction between CS Type and Contingency, *F*(1, 385) = 8.24, *p* = .004, *η^2^* = .02, BF_1_ > 10^5^. Most importantly, this interaction was qualified by the expected three-way interaction between CS Type, Contingency, and Measure, *F*(1, 387) = 4.73, *p* = .030, *η^2^* = .01, BF_1_ = 1.48. Contingency instructions moderated the CS Type effect on expectancy ratings, *F*(1, 385) = 14.19, *p* < .001, η^2^ = .04, BF_1_ = 95.79, but not on liking ratings, *F*(1, 385) = 0.01, *p* = .92, η^2^ < .01, BF_0_ = 8.83.^2^ Finally, we also observed a significant interaction between CS Type, Co-occurrence, Time, and Measure, *F*(1, 385) = 5.59, *p* = .019, *η^2^* = .01, BF_0_ = 1.80, indicating that, for Time 2 ratings, Co-occurrence moderated the CS Type effect on liking ratings, *F*(1, 385) = 4.58, *p* = .030, *η^2^* = .01, BF_0_= 1.10, but not on expectancy ratings, *F*(1, 385) = 0.64, *p* = .42, *η^2^* < .01, BF_0_ = 6.53. We did not observe a CS Type × Co-occurrence × Measure interaction for Time 1 ratings, *F*s < 0.03, *p*s > .88, *η^2^*s < .01, BF_0_s > 4.28.^3^

**IAT.** The 2 × 2 ANOVA on IAT scores from both experiments combined revealed only an effect of Contingency, *F*(1, 368) = 7.88, *p* = .005, *η^2^* = .02, BF_1_ = 5.56. IAT scores were significantly higher than zero when instructed contingency was low (*M* = 0.13, *SD* = 0.42), *t*(167) = 4.02, *p* < .001, *d* = 0.31, BF_1_ = 172.79, but not when it was high (*M* = 0.00, *SD* = 0.46), *t*(203) = –0.10, *p* = .92, *d* = 0.01, BF_0_ = 12.72. We did not observe any other effects, *F*s < 1.05, *p*s > .30, *η^2^*s < .01, BF_0_s > 5.65.

## Discussion

The observation that statistical contingency moderates expectancy learning but not evaluative conditioning is typically interpreted as support for the idea that these types of learning depend on distinct learning processes (Hofmann et al., 2010). We tested an alternative explanation according to which both types of learning depend on the formation of propositional beliefs but changes in liking reflect co-occurrence beliefs (i.e., that the CS and US have co-occurred) and changes in expectancy reflect contingency beliefs (i.e., that the CS is a reliable predictor of the US). In two studies, we set out to manipulate these beliefs by varying instructions about future stimulus presentations. More specifically, different participants received different instructions about the number of times that a CS+ would co-occur with an unpleasant US and the number of times the CS+ would be presented on its own. As predicted on the basis of the propositional account, the instruction-based manipulation of statistical contingency influenced US expectancy ratings but not ratings of US liking.

This main finding has a number of theoretical implications. First, it highlights that the differential impact of statistical contingency on evaluative conditioning and expectancy learning should not necessarily be seen as evidence for the existence of separate learning processes (e.g., two different association formation mechanisms as postulated by referential learning theory; Baeyens et al., 1992; Sweldens et al., 2010). Instructions are likely to have an impact via a single propositional learning process that can produce different types of beliefs about stimulus relations. The fact that an instruction-based manipulation of statistical contingency can also differentially affect changes in liking and US expectancy therefore fits well with the idea that both types of learning depend on propositional learning but differ with regard to the content of the beliefs that determine responding.

Second, it is not only the case that our results do not require multiple learning mechanisms, it is also difficult to see how they could be produced by multiple learning mechanisms. One might argue that instructions not only result in propositional beliefs but also in the formation of non-propositional associations (i.e., unqualified links between mental representations through which activation can spread; see Field, 2006, for such a proposal). Our results could then be explained if one assumes that changes in liking are mediated by co-occurrence-based associations whereas changes in US expectancy are driven by contingency-based propositional beliefs. Note, however, that this dual process explanation would imply that (a) expectancy learning is expelled from the domain of association formation models and (b) only instructions about co-occurrences lead to association formation. Alternatively, one might argue that instructions can somehow produce two types of associations based on two learning mechanisms, one that reflects co-occurrence information and drives changes in liking and one that reflects statistical contingency information and drives changes in US expectancies. However, such a post-hoc solution would intensify questions about the mechanisms via which instructions (and propositional beliefs more generally) influence associations (Mitchell et al., 2009). In sum, although it is possible to construct dual process accounts of our results, stringent post-hoc assumptions are necessary to make these accounts work. A more reasonable solution for dual process models of learning would be to concede that all effects of instructions about stimulus pairings are mediated by propositional beliefs. This would imply, however, that a second learning process (association formation) is required only to account for the effects of actual stimulus presentations (unlike to what is claimed by researchers such as Field, 2006, and Fazio, 2007). It also leaves unanswered the question of why such a second process needs to be assumed if the effects of instructions about stimulus presentations so often mirror the effects of actual stimulus presentations.

Third, the findings reported in this paper support single process propositional theories of learning. In general, these theories predict substantial similarity between the effects of actual stimulus presentations and the effects of instructions about stimulus presentations, provided that instructions carry the same information as actual events (De Houwer, 2009). This prediction has been confirmed in several experiments (e.g., Gast & De Houwer, 2013; Kurdi & Banaji, 2017; Mertens et al., 2018; but see Hu, Gawronski, & Balas, 2017, for an interesting dissociation in the context of evaluative conditioning). Of course, observing these similarities does not necessarily imply that the same (propositional) learning process is always responsible for both learning via instructions about stimulus presentations and learning via actual stimulus presentations. Nevertheless, it adds weight to the idea that propositional processes are important in many instances of learning. Our results also illustrate the predictive power of propositional models of learning. Until now, the propositional account of the differential impact of statistical contingency on evaluative conditioning and expectancy learning was purely post-hoc. Based on this post-hoc account, however, we predicted and confirmed that a similar dissociation can result from an instruction-based manipulation of statistical contingency.^4^

Our research also has a number of limitations. First, the dissociation that we observed does not necessarily imply that changes in liking depend on a different type of beliefs (i.e., about co-occurrence) than changes in US expectancy (i.e., about contingency). Our main result can be described as a single dissociation: a variable (i.e., instructed statistical contingency) that influences one phenomenon (i.e., changes in US expectancy) but not another (i.e., changes in liking). As is always the case with single dissociations, such a result might simply reflect a difference in sensitivity to the manipulation (e.g., Dunn & Kirsner, 2003). More specifically, it is possible that also changes in liking do depend on contingency beliefs (rather than only co-occurrence beliefs) but that they are simply less sensitive to those beliefs than changes in US expectancy. Nevertheless, even the latter conclusion would be interesting as such.

Second, we also hesitate to draw strong conclusions about the impact of the number of co-occurrences. In the present studies, neither changes in liking, nor changes in US expectancy were clearly affected by whether we told participants that the CS+ and US would be presented together 4 times or 8 times. We only observed an impact of the number of co-occurrences on liking when participants gave their ratings retrospectively (i.e., at Time 2). In hindsight, our manipulation might have been too minimal to produce strong effects. Perhaps a more extreme manipulation (e.g., 1 versus 20 CS-US pairings) would have produced a clearer difference. This issue also highlights another limitation of our studies: we only examined the effects of instructions about stimulus presentations but did not directly compare this with the effects of actual stimulus presentations. It is, for instance, possible that also a difference between 4 and 8 actual CS-US pairings would have had little impact within the current set up (e.g., in a procedure with only two CSs). More research is therefore needed to directly compare the effects of instruction-based and actual manipulations of the number of co-occurrences on evaluative conditioning and expectancy learning.

Third, also the IAT results are difficult to interpret. We added the IAT to capture more spontaneous evaluations of stimuli but had little reason to suspect differences between evaluative conditioning as indexed by the IAT and as indexed by evaluative ratings. Contrary to what we expected and unlike to what we observed for the evaluative ratings, the instruction-based manipulation of statistical contingency did have an impact on IAT scores. More specifically, participants exhibited an implicit preference for the CS+ over the CS– when they had received low contingency instructions but not high contingency instructions. Although this observation does not nullify the dissociation that we observed in the evaluative ratings, it does raise questions about the generality of this dissociation (e.g., it might only apply to rating measures) and the nature of the impact of statistical contingency on spontaneous evaluations.

Regarding the generality of the dissociation, it is interesting to note that – to the best of our knowledge – our study is the first to examine the effect of statistical contingency on spontaneous evaluations (also see Footnote 1). It would thus be interesting to examine in future studies whether a manipulation of statistical contingency via actual stimulus presentations also has an impact on IAT scores but not evaluative ratings. In fact, it would also be interesting to test the effect of (instruction-based and experience-based) manipulations of statistical contingency on spontaneous expectancies because those could also deviate from more controlled, well-reasoned expectancies (see De Houwer & Vandorpe, 2010, for a related idea, and De Houwer, Heider, Spruyt, Roets, & Hughes, 2015, for a measure of spontaneous beliefs).

Regarding the way in which instructions influenced IAT scores, it is surprising to see that the mean IAT score was positive in the low contingency condition (indicating a preference for the CS+ over the CS–) and close to zero in the high contingency condition (indicating similar liking of CS+ and CS–). We expected that participants would always like the CS+ less than the CS– because instructions stipulated that only the former co-occurred with the aversive scream (which is also what we observed in the liking ratings). It is unclear why IAT scores were not in line with this expectation. All variables in the study were fully counterbalanced, thus allowing one to interpret a zero score on the IAT as an equal preference for the CS+ and CS– and a positive score as a preference of CS+ over CS–.^5^ We thus refrain from drawing strong conclusions on the basis of the IAT results.

In sum, by demonstrating that the content of instructions about upcoming stimulus presentations differentially influence changes in liking and changes in US expectancy, we highlight that dissociations between learning phenomena do not necessitate the assumption of multiple learning processes. Our results are in line with the prediction that was derived from a single process propositional model but are difficult to explain upon the idea that multiple learning processes were involved. The results also reveal the potential of future research on the effects of (instruction-based manipulations of) statistical contingency and CS-US co-occurrence, including the effects of those manipulations on spontaneous evaluations and beliefs.

## Data Availability

The pre-registration, raw data, experimental and analytic scripts are available at https://osf.io/f9jn6/.

## Footnotes

Although we are not aware of any previous studies on the effects of statistical contingency in which implicit evaluations were measured, there have been several studies showing that an extinction procedure does not influence conditioned changes in implicit evaluations (e.g., Gawronski, Gast, & De Houwer, 2015; Hermans et al., 2002; Vansteenwegen et al., 2006). Note, however, that a manipulation of statistical contingency involves CS only (or US only) trials that are intermixed with CS-US pairings and thus refers to the acquisition of learned behavior, whereas an extinction procedure involves CS only trials that are presented after the CS-US pairings and thus refers to changes in behavior after it has been acquired. We therefore do not discuss in detail studies on extinction in evaluative conditioning and expectancy learning.

We also performed two ANOVA’s on standardized Time 1 and Time 2 US expectancy and liking ratings for Experiment 1 and Experiment 2 separately. This revealed the CS Type × Contingency × Measure interaction for Experiment 1, *F*(1, 177) = 3.67, *p* = .058, *η^2^* = .02, but not Experiment 2, *F*(1, 204) = 1.32, *p* = .25, *η^2^* = .01. Hence, the conclusion that instructions *differentially* affected liking and expectancy was not supported by the ANOVA of the rating data of Experiment 2. Nevertheless, in both experiments, Bayes Factors provided strong evidence for an effect of contingency information on US expectancy ratings as well as strong evidence for the absence of such an effect on CS liking ratings. Together with the CS Type × Contingency × Measure interaction in Experiment 1 and in the combined analyses, we thus feel safe in concluding that contingency information does indeed have a different impact of US expectancy ratings than on CS liking ratings.

ANOVA’s on standardized Time 1 and Time 2 US expectancy and liking ratings for Experiment 1 and Experiment 2 separately revealed a CS Type × Co-occurrence × Time × Measure interaction for Experiment 2, *F*(1, 204) = 3.86, *p* = .050, *η^2^* = .02, BF_0_ = 1.10, but not Experiment 1, *F*(1, 177) = 2.21, *p* = .14, *η^2^* = .01, BF_0_ = 3.39.

Note that current propositional models do not specify why evaluative conditioning would depend primarily on co-occurrence beliefs whereas expectancy learning depends on contingency beliefs. On the one hand, one could argue that stimulus expectancy can be logically inferred from contingency beliefs (but see De Houwer, Vandorpe, & Beckers, 2007). On the other hand, there seems to be no logical reason why the liking of a stimulus should depend primarily on co-occurrence beliefs. It is, however, important to realize that propositional models focus on learning (in the sense of the acquisition of knowledge) rather than behavior (i.e., the processes via which knowledge influences behavior; Mitchell et al., 2009). Hence, they do not necessarily imply that behavior will change only when it can be rationally inferred from the available propositional beliefs (see Moors, 2014, for an excellent discussion and Van Dessel, Hughes, & De Houwer, in press, for the proposal that irrational behavior might result from automatic inferences on the basis of propositional beliefs).

The only exception was that the order in which the CSs were presented during the rating phase was linked to response assignments of the CSs in the first block of the IAT. That is, the CS that was presented first during the rating phase was always assigned to the left response during the first block of the IAT. However, it is difficult to see how this could influence the IAT scores in a systematic manner.
